# Global estimates of country health indicators: useful, unnecessary, inevitable?

**DOI:** 10.1080/16549716.2017.1290370

**Published:** 2017-05-22

**Authors:** Carla AbouZahr, Ties Boerma, Daniel Hogan

**Affiliations:** ^a^ CAZ Consulting, Geneva, Switzerland; ^b^ Department of Information, Evidence and Research, World Health Organization, Geneva, Switzerland

**Keywords:** Bringing the indicators home: Country perspective on the utility of global 40 estimates for health indicators (WHO), Global health estimates, data users and uses, health statistics, modelling, ethics, capacities, country health information systems

## Abstract

**Background**: The MDG era relied on global health estimates to fill data gaps and ensure temporal and cross-country comparability in reporting progress. Monitoring the Sustainable Development Goals will present new challenges, requiring enhanced capacities to generate, analyse, interpret and use country produced data.

**Objective**: To summarize the development of global health estimates and discuss their utility and limitations from global and country perspectives.

**Design**: Descriptive paper based on findings of intercountry workshops, reviews of literatureon and synthesis of experiences.

**Results**: Producers of global health estimates focus on the technical soundness of estimation methods and comparability of the results across countries and over time. By contrast, country users are more concerned about the extent of their involvement in the estimation process and hesitate to buy into estimates derived using methods their technical staff cannot explain and that differ from national data sources. Quantitative summaries of uncertainty may be of limited practical use in policy discussions where decisions need to be made about what to do next.

**Conclusions**: Greater transparency and involvement of country partners in the development of global estimates will help improve ownership, strengthen country capacities for data production and use, and reduce reliance on externally produced estimates.

## Background

The era of the Millennium Development Goals (MDGs) was characterized by growing demand for statistics to inform health planning, monitoring and accountability. Demand is likely to accelerate further with the Sustainable Development Goals (the SDGs) that encompass more indicators and require more detailed disaggregation than the MDGs. In response, the supply of statistics has increased, with innovative methods developed to collect and analyse data and more funding for data collection programmes such as the USAID-funded Demographic and Health Surveys (DHS) and the UNICEF-funded Multiple Indicator Cluster Surveys (MICS). At the same time, there have been vast increases in the production of health estimates by researchers, academics and international agencies since the 1990s.

**Figure 1.**
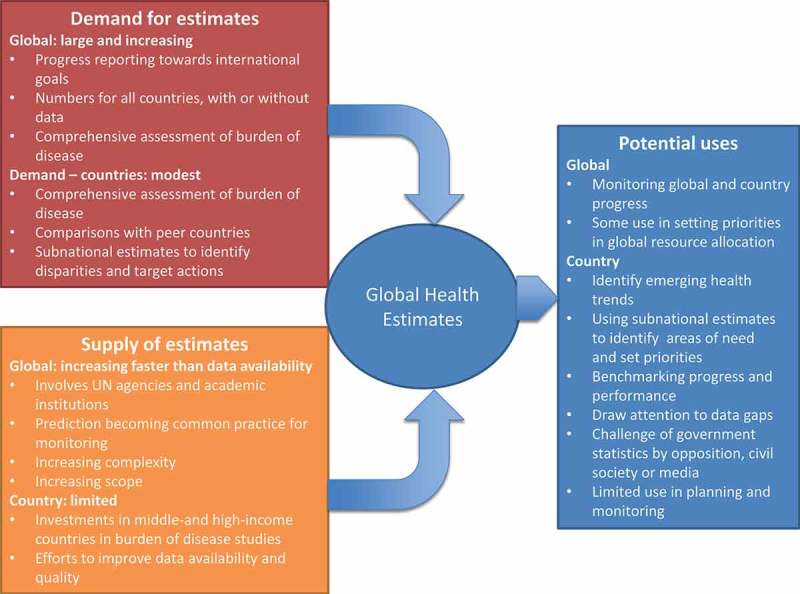
Supply and demand interactions in the production and use of global health estimates.

The resulting abundance of quantitative information on health is manifest in statistical publications regularly issued by the World Health Organization (WHO), other health-related agencies at global and regional levels, and academic groups. The history of how this has come about is illuminating and raises questions as to who generates the numbers and who are the users at global and country levels. This paper offers an overview of these developments and sets the context for this series of country and technical papers on the implications of global health estimates for countries and for development partners. We describe the evolution of global health statistics since the late 1990s and the emergence of the science of health estimates from the perspectives of supply and demand (Figure 1). We propose ways of reconciling the concerns of countries on the receiving end of health estimates produced by international agencies with the desires of development partners to have at their disposal reliable and up-to-date information on progress towards agreed goals and targets.

## What are global health estimates?

Available data for health indicators typically suffer from a number of limitations: inadequate measurement techniques, differences in definition, incompleteness of coverage, missing values, sampling error, lack of timeliness and errors in the reporting or coding of the collected information. The challenge is to make the best possible use of available data despite their weaknesses by resolving these issues using a variety of methods, in other words by producing estimates. How to do so in ways that are simultaneously technically sound, meaningful for users and acceptable for decision makers is the subject matter of this series of papers.

In the general discourse, there is a tendency to use ‘data’, ‘statistics’, ‘indicators’ and ‘estimates’ interchangeably. In this paper we use the term ‘global health estimates’ to refer to values for health indicators developed for multiple countries (and usually by entities with an international remit), typically by synthesizing multiple data sources with a statistical or mathematical model. This contrasts with ‘data’, which we refer to as empirically measured values derived from a particular data source such as a sample mean computed from a household survey. However, there is no unambiguous line that separates data from estimates.

In practice, communicating all that goes into generating estimates from country data is a major challenge. Conceptually, it can be helpful to think of three general processes that are often undertaken to derive estimates from data:

- *Direct calculation*. This typically involves standard calculations to convert the information in the original data source into the quantity of interest or indicator. Examples include computing a national prevalence estimate of family planning coverage from a survey dataset based on survey participant responses and sampling weights, or using conventional demographic methods to compute an estimate of the under-five mortality rate from full birth history data collected in a survey.
- *Adjustment for potential sources of bias*. As mentioned already, sampling and non-sampling errors abound in many data sources, and rigorous estimation efforts will seek to identify biases and, if any are identified, adjust for them to produce a more accurate estimate. Examples include redistributing ill-defined causes of death when deriving cause of death estimates from vital statistics data, smoothing noisy data to produce an estimate of the underlying trend in a mortality rate, and accounting for known underreporting of maternal deaths when estimating maternal mortality ratios.
- *Prediction of missing data*. Producers of global health estimates routinely generate estimates for all countries across time, even when data from many countries or time periods do not exist. This often involves fitting statistical models to countries with data, typically including other variables like gross domestic product (GDP), and then predicting an estimate for countries or time periods without data. These prediction activities can range from the fairly modest, for example interpolating a trend line for intervening years between multiple surveys in a country, to the more heroic, such as extrapolating forward several years beyond the last observed data point in a country (‘forecasting’) or predicting values for a country with no data at all (‘farcasting’).

Interpreting global health estimates for different purposes requires an understanding of the extent to which these three different processes have been used. However, this can be a daunting task in many cases, as complex statistical models may be used to adjust data inputs for bias while simultaneously predicting missing data, resulting in an estimate with a fuzzy methodological footprint.

## Demand for global estimates

When the United Nations (UN) system was established following the Second World War, the need to have basic data on member countries, including population size and change, became quickly apparent. From 1950 the United Nations Population Division (UNPD) has produced regular estimates for all countries of population by age group and sex, fertility and mortality. To produce the estimates, demographic data from all sources are evaluated for completeness, accuracy and consistency and adjusted as necessary using methods that have become more sophisticated over time [1]. Initially, the estimates were revised on a quinquennial basis; more recently revisions have been published biennially. Each revision takes into account all the country empirical datasets used for previous revisions and additional datasets and information that have become available since the last revision.

In the health arena, demand for better data grew out of global declarations signed by heads of state starting in 1978, with the Alma Ata Declaration on Primary Health Care. During the 1980s and 1990s a series of global conferences was organized by the UN on various aspects of health and development, including child survival (1990), population (1994)^1^ ^1^Population conferences have taken place on a decennial basis since 1954, initially focussed on population growth and change. The 1994 conference represented a turning point bringing together health and population as part of a broader development agenda. and food security (1996). The annual International AIDS Conferences from 1985 stimulated demand for better data on the spread of the epidemic. The UNICEF Summit for Children in 1990 led to production of annual estimates of child mortality for countries, published in the UNICEF report ‘State of the World’s Children’. The 2000 Millennium Declaration and associated MDGs represented a culmination of these conferences, bringing together into one framework their main goals and targets and setting out a time-bound monitoring schedule.

From the outset, it became apparent that there were serious deficiencies in the availability and quality of data reported by countries to WHO, UNICEF, UNFPA (United Nations Population Fund) and other international agencies [2,3]. Routinely reported data based on the service provision-related activities of health programmes were incapable of adequately reflecting the health status of populations as a whole. In response, innovative technical approaches were developed to measure key indicators of fertility and mortality through sample surveys. These methods were incorporated into the DHS and MICS. Over time, these and similar survey instruments have become the major vehicle for generating an understanding of health status, behavioural and environmental risk factors, and health service utilization, especially in low- and middle-income countries.

However, not all countries conduct surveys on a regular basis. Furthermore, survey methods have some limitations for global and country monitoring. For example, the survey-based estimates relate to a period in the past, have uncertainties associated with sample size limitations and respondent errors, and generate indicator values for national or major subnational areas but not for local administrative areas. Thus, while large survey programmes have alleviated the pressure for data, they have not removed it. Countries and development partners continue to demand timely, complete, accurate statistics that are comparable over time and across geographies and other stratifiers.

Demand will inevitably grow in the SDG era. The 17 SDGs comprise 169 targets and 230 indicators, including an overarching health goal that has 13 targets and around 34 health-related indicators (at last count) that need to be monitored. Furthermore, a strong focus on equity means that indicators should be capable of disaggregation along multiple dimensions. In response, the science and practice of model-based estimation will continue to evolve, with new SDG estimates already published in 2016 [4,5].

## Rationale for global health estimates

**Figure 2.**
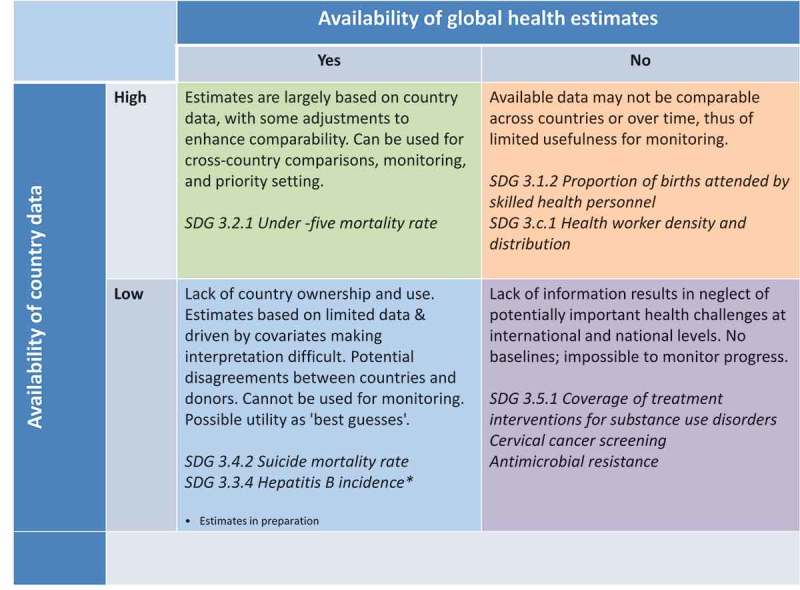
Relationship between country data availability and quality and presence of global health estimates.

There are many valid reasons for developing global health estimates, which can be summarized briefly as the drive to fill data gaps and ensure completeness of information for all countries; comparability of data over time and across countries; currency or immediacy of indicator values; independence and objectivity; and cost containment (Box 1). Estimates are also a response to the need for a comprehensive picture of the health situation and trends across multiple diseases and conditions in order to guide country priority-setting. The utility of estimates and the relative importance of each of these drivers will depend on the extent and quality of available country data (Figure 2).

Estimates developed by global entities offer important assurance of completeness and comparability. They generate historical time series as well as forward-looking projections so that indicator values have currency and are relevant to today’s situation, rather than to the recent past. This desire for immediacy is accentuated by recent trends towards performance-based funding, a culture of wanting to see results now and using the estimates to disburse funds, satisfy donors and raise more funds.

A related issue is the desire to ensure that reported data are developed in an independent and objective manner, free from perceived or real political interference. This is key for accountability. An additional driver of demand, not to be underestimated, is the need to achieve all of the foregoing in a manner that is relatively inexpensive. Notwithstanding efforts to strengthen country health information and statistical systems, the fact is that it is far less expensive to generate data centrally using technical expertise and computing power than to spend time, effort and resources in the uncertain and long-term business of supporting capacity development for data production and statistical analysis and interpretation in the field.

## WHO and health estimates: an evolving relationship

The WHO took some time to get involved in producing global health estimates. Following its establishment in 1946, the Organization disseminated health statistics under its constitutional mandate to provide ‘epidemiological and statistical services’ [6]. The publication *World Health Statistics Annual*(published 1957–1998) brought together data reported by countries on key indicators such as infant mortality and causes of death. These were considered the official health statistics of countries. Because the figures reported by many countries were far from complete or reliable, and some countries reported no figures for key indicators, the publication used italic script for reports considered uncertain and left blank spaces when no acceptable data were available. The UN *Demographic Yearbook* still uses a similar approach for population and vital statistics as of today [7].

Over time, with the realization that reliance on country reporting was inadequate for making a global assessment of the state of the world’s health, the WHO has become increasingly engaged in the production of global health estimates, working together with other UN agencies and with academia to fill data gaps and to compensate for the inadequacies of country-reported figures. Since 2005, the remodelled WHO flagship publication *World Health Statistics*, and its related web portal, offer regular statistical reports on the current situation and trends for priority health issues [8]. In today’s publications, users will find few blank spaces to indicate missing values or italics indicative of unreliable data. The 2016 edition of *World Health Statistics* includes indicator values for 34 health-related SDGs for all countries [9]. Many of the numbers are model-based estimates based on limited inputs of country data.

The estimation process applied may vary from country to country and from indicator to indicator as a function of the underlying empirical data available and choice of statistical model. Yet country indicators are often presented in the same way regardless of the underlying data sources and estimation method. From a user perspective, it is not readily clear what kind of number is being presented in any specific case [10]. It takes a dedicated reader to refer back to the explanatory notes and even then enlightenment may not be provided. In recognition of this problem, WHO has used a colour-coding scheme for some of its estimates to indicate the availability of underlying data. While certainly an advance, this falls far short of what users need to interpret individual country indicators.

**Figure 3.**
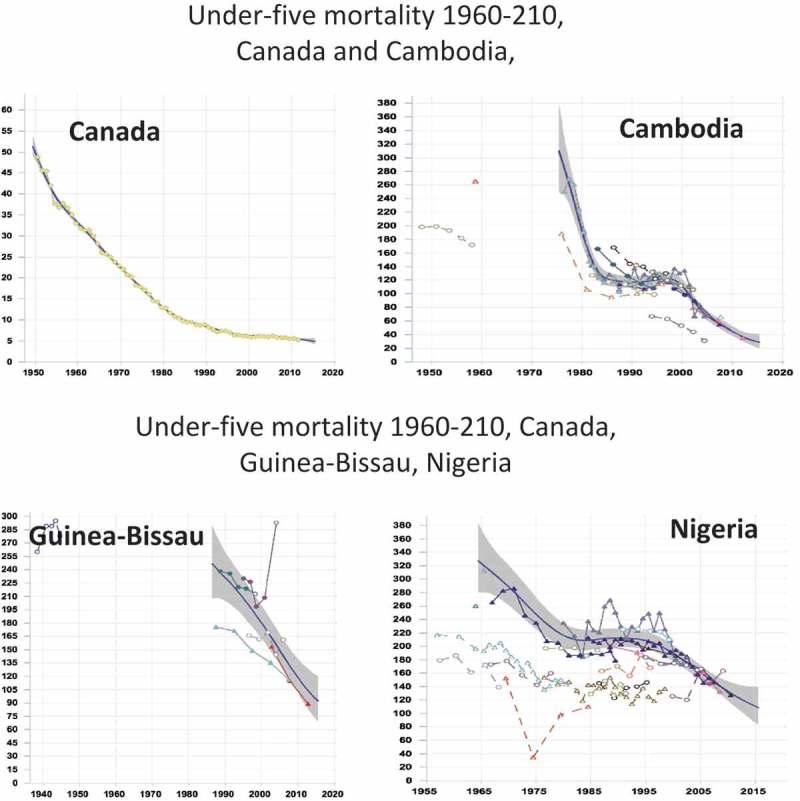
Estimated trends in under-five mortality, Canada, Cambodia, Guinea-Bissau, Nigeria.

Investigative readers can access detailed methodological information underlying the country estimates through the web sites of WHO and other agencies involved in the estimation process. Thus they can find out that with regard to an indicator like child mortality rates, there are important differences between, for example, Canada, Cambodia, Guinea-Bissau and Nigeria in the richness and quality of the underlying empirical database, the contribution of statistical modelling, and the degree of uncertainty of the indicator values. Canada’s estimates are computed from a complete and comprehensive vital registration system, requiring little adjustment and yielding very narrow uncertainty ranges. By contrast, the data for Cambodia, Guinea-Bissau and Nigeria are derived using a mix of adjusted census and household survey data that are synthesized with the statistical model. The availability and consistency of survey and census data vary considerably between the three countries: Cambodia has multiple datasets with a high degree of consistency in recent periods whereas Guinea-Bissau has few data and Nigeria has many datasets with considerable inconsistency (Figure 3 [11]). So statements about level of mortality in children under 5 years of age have varying degrees of precision. Similar considerations apply to other mortality indicators and to indicators of disease incidence and prevalence.

When global agencies use estimates for country reporting they acknowledge – somewhat obliquely – that national governments may not accept and use the estimates for national purposes. The WHO estimates are described as ‘official WHO figures’ with the caveat that they ‘should not be regarded as the nationally endorsed statistics of Member States’ [12]. In other words, the estimates are seen as external to countries themselves, primarily designed to meet the WHO mandate of providing information in relation to public health.

As the country papers in this series demonstrate, country decision makers may look askance at the usurpation by global agencies of countries’ responsibility for statistical reporting. And while the WHO affirms its desire to ‘reconcile data provided at the global level with the data published by national statistical authorities’ [13], the mechanisms for doing so are insufficiently developed, an issue we take up later in this paper and that is discussed in the other papers in this series.

## Differing perspectives on global health estimates

### International users of estimates

Global health estimates are extensively used by entities involved in developing global declarations that include commitments to the achievement of specific goals and targets within a specified time frame. The most obvious use is to track progress towards international goals (Box 2). Perhaps the most assiduous users of global health estimates are actually those who produce them, namely international development agencies and academic institutions such as the Institute for Health Metrics and Evaluation (IHME).

Many donors, funds and foundations use global estimates to track progress towards their own project- or initiative-specific goals and results-based indicators, and to evaluate impact because they perceive that country-reported data lack an acceptable degree of quality, timeliness and comparability. While global estimates help donors make more informed decisions, reliance on them to allocate funding may have unintended effects. In an age where numbers prevail, the availability of seemingly robust estimates from trusted institutions legitimizes the prioritization encapsulated in global frameworks such as the MDGs and SDGs [14]. Convergence of donor funds around a few issues can result in displacement within country budgets, and distortion or crowding out of other country-determined priorities [15]. Donors and development agencies armed with global estimates from respected agencies or academic institutions are less likely to arrive at the same set of priorities as countries using their own empirical data.

International users recognize the advocacy value of estimates and use them to underpin high-level visibility for a particular health challenge. This can lead to potential conflicts of interest. For example, when estimates appear to show improvements in certain indicators, programme managers and advocates may fear loss of attention for ‘their’ priorities. Funding issues often fuel debate about the relevance of the estimation process.

The advocacy machine drives another global user, the media. Journalists tend to favour numbers produced by international organizations over those produced by national governments, regarding them as more authoritative. They also want to make headlines with comparable data that are very recent. In general, only a predicted estimate can fulfil that need on global and regional scales.

### Country views

The readiness of international partners to use global health estimates contrasts with the reticence with which they are often received at country level, particularly by senior decision makers, including Ministers of Health, who are held responsible for country performance with respect to international health-related goals. Many countries have been mistrustful of global estimates, especially when they diverged considerably from country-reported data. Such resentment emanates not only from the details of methods, but also, and with particular force, from political considerations. Notwithstanding the often large uncertainty around many modelled estimates, political opponents, media and civil society may use them to challenge government statistics and policy responses. While transparency and debate are generally to be encouraged, the presentation of global estimates as objective and independent does not necessarily mean that they have a monopoly on the ‘truth’.

Country perspectives tend to be coloured by the manner in which estimates are developed and their degree of involvement in and understanding of the process (Box 3). For example, there was widespread concern in 2000 when the WHO developed estimates of health system performance and used these to rank countries [16,17]. Criticisms focused not only on the details of the technical estimation methods and lack of underlying data but also on the absence of country involvement and consultation.

Policy makers rarely have either the inclination or the skills to develop an understanding of the estimates and will turn to locally generated estimates, which they perceive to be more ‘real’ than extraneous figures derived using hard-to-follow statistical techniques. In practice, locally derived estimates are more likely to be used for highlighting differentials and supporting local decision-making.

It was to give voice to countries that the WHO convened, in June 2015, an intercountry meeting on the utility of global health estimates [18]. Some of the key issues that emerged from that meeting and subsequent discussion include the following.

#### Input data manipulation

Concerns arise about the way country empirical data are adjusted as part of the estimation process. Even when data derive from established sources such as surveys, they are often adjusted to such an extent that they are hard to recognize. The detail of such manipulations is rarely described in sufficient detail, yet should be understandable and reproducible by country technical staff.

#### Divergent estimates

Acceptance of estimates by country decision makers is put at risk when different estimation techniques give rise to different values for the indicators. There are two types of divergences:

- *Differences between producers*. Estimates from different groups (for example from UN agencies and academic institutions) differ from each other and also differ from country data [19,20]. How is a user – whether at country or international level – to decide which estimate is closer to the ‘truth’? Such differences are an inevitable result of the differences in methods, which evolve over time but they risk fostering policy inactivity if the reasons for the changes are not made clear. From a country perspective the differences are difficult to explain to users who lack the skills to determine which approach is more appropriate for the country.
- *Shifting baselines*. Because every estimation revision involves a complete reassessment of input data and often of statistical modelling approaches, the whole time series for each indicator is modified, producing a new series at baseline and over time. Changing historical series can make target-setting difficult. A formal government process may result in adopting a particular target based on current estimates, only to find a few years later with a new estimate that the target either is impossible to reach or has already been met. Shifting baselines not only cause confusion about the true extent of progress but also may result in political fallout if annual rates of change reported to global processes such as MDGs are significantly modified, as noted in the paper from Brazil.

#### Failure to identify predictions

Further confusion arises when health estimates are published for the current year and not clearly labelled as predictions based on extrapolations of trends and use of correlations. Monitoring progress with projections defeats the purpose of monitoring, which is to detect the results of changing circumstances or programme interventions over time. Predicted statistics cannot detect changes due to recent adjustments in policy or sudden deterioration in circumstances and so should not be used for this type of monitoring and assessment but it is widely done [21]. Moreover, in countries with no data, it makes little sense to use global health estimates to judge progress on a particular indicator, as the trend estimate is entirely determined by statistical modelling assumptions.

#### Uncertainty about uncertainty

Whereas technical experts emphasize the uncertainties surrounding global estimates, experts in countries find this a difficult concept to convey to policy makers and the media who demand a single figure upon which to base a policy decision. Better ways of conveying uncertainty are needed that can be readily explained to non-experts, but good examples are few and far between.

#### Insufficient consultation

A frequently cited problem is the lack of consultation with countries by global agencies producing estimates [22]. Country decision makers and technical experts have a deep understanding of the data sources and health system context in their countries, and have to make important policy decisions. They therefore need to understand, interpret and judge health estimates before they can responsibly use them to change public policy and reallocate resources. Lack of consultation may give an impression that countries are simply passive providers of datasets for analysis by others [23,24]. No matter how complex the statistical modelling tools, and persuasive the data visualizations, unless country decision makers or trusted country experts are themselves able to understand and interpret the estimates, few will have confidence in their accuracy and actually use them [25]. Although such consultation processes can be time-consuming and demanding from the viewpoint of those producing estimates, they can also be positive, especially when countries take on an active role, providing new data and engaging in the development of the methods. Unfortunately, the time constraints under which estimates are produced – generally to meet external deadlines such as an MDG or *World Health Statistics* report – militate against such inclusive collaboration and consultation.

Attitudes to global health estimates are by no means uniformly negative. Many countries acknowledge the utility of estimates for benchmarking national progress. Comparisons over time and across subnational boundaries can help secure continued support for successful programmes and stimulate action for less successful ones. Technocrats in countries that perform badly compared with countries with similar socioeconomic profiles can use global rankings to needle politicians into action, improving both data systems and programme implementation (historically in Chile, more recently in South Africa and Iran). The most positive views of global estimates are expressed by countries such as Brazil [18], Chile [26], Mexico [18], South Africa and Thailand [22] that have been stimulated to bring the estimation process home by taking on the global estimates and producing their own country estimates based on country data and nationally determined assumptions.

Examples of processes that successfully combine technical soundness with a satisfactory process and country ownership do exist, for instance in HIV estimation as described in the paper by Mahy et al. in this series [27]. A series of regional workshops, supported by user-friendly estimation tools with a clear link between input data and resulting estimates, has over time contributed to the development of national capacity to produce estimates in most countries; these are refined in an iterative process together with technical specialists from UNAIDS and collaborating agencies and academics. The process has taken many years and many millions of dollars to develop; it has unquestionably led to better data at national and subnational levels, as well as much better use of data by countries. Though the process has had important benefits nationally, it was made possible because international funders for close to two decades overwhelmingly prioritized HIV, and there was high-level policy support for efforts to improve the availability and quality of country-based data. Similar efforts in the field of estimation of child mortality and maternal mortality are on-going but with considerably fewer resources than HIV, which means that not enough people and countries can be reached on a recurrent basis.

## The supply side: a booming business

### Diversification of partners

A lesson learnt from over 20 years of global health estimates is that demand is never satisfied. Indeed, increased supply of estimates seems to stimulate ever more demand. As data analysts come up with more clever ways of making the most of bad or missing data, demand escalates for more detailed, disaggregated, frequent and timely estimates.

As demand for health estimates has expanded, more entities, researchers, academics and international agencies have become involved, sometimes working in collaboration with the UN agencies, and sometimes working independently. One example of collaboration was the Child Health Epidemiology Reference Group (CHERG), a group of technical experts convened to assist WHO and UNICEF with the goal of providing global technical leadership in the development and improvement of epidemiologic estimates for children under age five [28]. Similar independent advisory and technical reference groups exist in other measurement areas, including HIV/AIDS, tuberculosis, malaria, maternal health etc. The existence of such groups not only provides the UN system with valuable technical expertise on which it can draw but also provides a degree of independence and objectivity, attributes that some have criticized as missing within the UN system [29].

In 2007, the world of health estimation entered a new phase with the establishment of the IHME, based at the University of Washington in Seattle, USA, with funding from the Bill and Melinda Gates Foundation. The IHME generates estimates and projections for a wide array of health indicators and often highlights differences with estimates produced by UN agencies such as the WHO and the UNPD. A major focus has been a systematic effort to quantify the comparative magnitude of health loss to diseases, injuries and risk factors by age, sex and geography over time around the world, the Global Burden of Disease (GBD) [30,31].

### From single indicators to big picture

Initially, estimation efforts focused on specific summary indicators such as population, fertility, life expectancy and mortality due to high-priority diseases and conditions. However, global health estimates have also been developed to quantify for all countries the burden of mortality and disease due to major causes of death and disease. The first such estimates were produced by researchers at Harvard University and the WHO for the 1993 World Development Report, *Investing in Health* [32], and were followed by annual updates by the WHO during 1999–2002 and four yearly updates from 2004. Since 2012 the IHME has collated and analysed data on premature death and disability for more than 300 diseases and injuries in 188 countries, by age and sex [33]. The core of the endeavour is a huge database of country health, mortality and covariate data, which are used as inputs to statistical models that generate global health estimates that are intended to be internally consistent and comparable across countries and over time.

Despite its many conceptual strengths, the GBD has been criticized on grounds of complexity and non-reproducibility [34–36]. The adjustment of data during the estimation process, which involves a multi-step process by which data quality is ‘assessed and enhanced’, is difficult to replicate even by other skilled and well-resourced technicians, making it hard to understand how the adjustments have affected the initial data inputs [37]. It is the extent of adjustment of the country data inputs that is most often a source of concern among users, particularly those at country level who are faced with the challenge of explaining the different figures to policy makers. The use of sophisticated mathematical models that require substantial computing resources to run may also contribute to a sense of inaccessibility of GBD estimates for users.

The GBD estimates can be useful for country policy formulation when used in the aggregate to paint an overall picture of mortality in a country. Their utility is less apparent when the individual cause-specific indicators are lifted out of this broad context, and presented as the ‘truth’ on the grounds that they derive from a consistent comprehensive model. Yet such models involve multiple adjustments to individual data series, ‘squeezing’ to accommodate estimates of the overall mortality envelope, and extensive prediction of missing data.

The outputs of GBD exercises are widely disseminated through peer-reviewed journals and special reports, but these do not provide the kind of detail of the methods that would enable an interested and technically competent reader to follow and reproduce them. Moreover, such technical publications are rarely read or understood by policy makers who are faced with the day-to-day challenges of resource allocation and accountability.

Several high-income countries and some middle-income countries such as Thailand have conducted national burden of disease exercises and have been able to focus on the application of the methods to their own circumstances rather than getting involved in disputes about particular indicator values [22]. Even when there is insufficient capacity to conduct a comprehensive national burden of disease exercise, countries in health transition such as Bangladesh and Ghana have applied the methods using country data to highlight emerging health challenges such as noncommunicable diseases (NCDs), violence, mental health and road traffic accidents.

Despite the criticisms, in general, global estimates of overall burden of disease distributions are more readily acceptable to policy makers than estimates for particular, high-priority indicators such as child or maternal mortality. Few Ministers of Health will face political retribution based on overall disease and disability distributions but failure to achieve reductions in maternal mortality or to control malaria can spell the end of a political career. Moreover, there is little information against which to compare the estimates in developing countries and so there are few grounds for dispute.

### The new frontier – estimates for small areas

The MDG era was characterized by the generation of health estimates at the macro level of the national and international community. This national-level focus was justly criticized on the grounds that it rendered invisible differentials in health status and health care utilization among disadvantaged groups and between geographic areas within countries. National-level estimates are of limited value for policy and planning at local level where many resource and managerial decisions must be made. Country policy makers often demand subnational data to guide the allocation of resources and assessment of performance.

For several years already, users have pointed out that national averages are insufficient to track country progress. Equity lies at the heart of the SDGs and is key to the achievement of Universal Health Coverage (UHC). The SDG emphasis on ‘leaving no-one behind’ will further motivate data analysts to come up with methods for generating estimates to support the spatial allocation of health resources within a country. SDG Target 17.18 specifically calls for disaggregation of data by various stratifiers including income, sex, age, race, ethnicity, geographic location, disability and migratory status.

The new frontline of methodological development that will vastly multiply the supply of estimates is subnational estimates. The IHME and others have begun to use advanced methods, such as Bayesian geospatial modelling, to also obtain small area estimates that can be aggregated to administrative units such as districts. Subnational estimates derived using such approaches may be helpful to countries by identifying areas of particular deprivation and drawing attention to the need to collect and analyse more empirical data in order to ascertain and respond to local priorities. However, given the limited availability of data, the more detailed the disaggregations, the more uncertain the resulting modelled estimates are likely to be, rendering inappropriate their use for monitoring progress and performance.

It remains to be seen if globally produced subnational estimates empower national- and local-level decision makers or if, instead, international donors and organizations use them to dictate priorities and ever-finer terms for how programmes should be implemented [38]. And subnational estimation raises ethical concerns around the risk of identification of subgroups or individuals as examined in the following section [39].

#### Estimates and ethics: a neglected perspective

Global health estimates are powerful. And where there is power, there is a need for ethics. The lively debate around the methodological aspects of global health estimates has not been matched by examination of the ethical concerns that they raise. Because global health estimates are used to underpin much of the public health discourse, particularly at global level, the ethical implications of decisions based on estimates, particularly those that are developed outside the country context, need to be made explicit and addressed.

The UN’s *Fundamental Principles of Official Statistics* includes professional ethics (at both individual and institutional levels) as one of its 10 fundamental principles [40]. Although there is no direct mention of global health estimates, several ethical and legal principles are of particular relevance, including transparency, trustworthiness, ownership and confidentiality.

Transparency involves keeping country stakeholders informed about how their data are used to construct estimates, how estimates can be used to make policy decisions and also the limitations of global estimates for national and subnational policy and planning. It also implies making input data and analytical methods accessible to qualified researchers in countries and internationally to independently verify, replicate and improve epidemiological estimation across multiple diseases and conditions [10].

Transparency is essential for building trust in the way estimates are produced and used for health policy and planning. If estimates are not trusted they will not be used and decision-making may be driven by inaccurate or flawed information. When estimates from different sources yield conflicting information, or there is more than one interpretation of a single set of findings, or if they are misleading or incorrect, a transparent and balanced decision-making process will be crucial to agree on the best course of action, remedy deficiencies in the estimates and prevent particular persons, groups or agencies from unilaterally imposing their view.

An important ethical consideration relates to ownership of the multiple sources of data used to generate estimates. These datasets may be drawn from country administrative sources such as birth and death registers, disease surveillance, health insurance databases or health facility reporting as well as from household surveys and censuses. The WHO has privileged access to such data sources through its constitutional mandate and the monitoring activities of various health programmes, and participates in data sharing with partners, including academia, on a more or less formal basis. Yet in principle, different entities could claim ownership to such information, including individuals, healthcare providers, disease registries, health insurance plans, funding agencies, research institutions and government agencies [41]. Although the benefits of data sharing for public health are well-known, all entities that deal with such data need to put in place, and abide by, policies to maximize the common benefits and minimize the risks to individuals, to governments and to agencies that use the data to come up with global and country estimates. Currently, few countries have an on-going public discourse or adequate policy and legal frameworks for data ownership and stewardship [42].

Although the data inputs to statistical estimation processes are usually aggregated, there are risks that the identity of individuals or particular administrative areas could be deduced, especially when data refer to small numbers or to defined geographical areas with small populations. This risk will grow more acute as the estimation business moves into the development of subnational and disaggregated statistics. In the era of big data from e-health [43] and m-health [44] innovations, interoperable databases, mobile phones, social media, geolocation devices and data triangulation between varieties of data sources, the ability to ensure anonymity is becoming increasingly challenging.

## The way forward

Out of the debates and discussions around global health estimates have emerged good practice recommendations for users and producers in countries, donors, international agencies and academia [45,46]. Among these are the GATHER (Guidelines for Accurate and Transparent Health Estimates Reporting) principles [47]. These consist of a checklist of 18 items that must be reported within each study publishing global health estimates, so that users may make an assessment of the quality of the estimate (Table 1). GATHER includes requirements for disclosing which data are used to calculate estimates, and for making them available to others. It also includes a requirement to disclose how the computer code used to crunch the numbers can be accessed, making it possible for others to reproduce estimates, making them more robust. A baseline review of the current status of adherence to the GATHER principles is one of the papers in this series [48].

**Table 1.** Checklist of information that should be included in new reports of global health estimates.

Item #	Checklist item
Objectives and funding	
1	Define the indicator(s), populations (including age, sex, and geographic entities), and time period(s) for which estimates were made.
2	List the funding sources for the work.
Data inputs	
*For all data inputs from multiple sources that are synthesized as part of the study:*	
3	Describe how the data were identified and how the data were accessed.
4	Specify the inclusion and exclusion criteria. Identify all ad-hoc exclusions.
5	Provide information on all included data sources and their main characteristics. For each data source used, report reference information or contact name/institution, population represented, data collection method, year(s) of data collection, sex and age range, diagnostic criteria or measurement method, and sample size, as relevant.
6	Identify and describe any categories of input data that have potentially important biases (e.g., based on characteristics listed in item 5).
*For data inputs that contribute to the analysis but were not synthesized as part of the study:*	
7	Describe and give sources for any other data inputs.
*For all data inputs:*	
8	Provide all data inputs in a file format from which data can be efficiently extracted (e.g., a spreadsheet rather than a PDF), including all relevant meta-data listed in item 5. For any data inputs that cannot be shared because of ethical or legal reasons, such as third-party ownership, provide a contact name or the name of the institution that retains the right to the data.
Data analysis	
9	Provide a conceptual overview of the data analysis method. A diagram may be helpful.
10	Provide a detailed description of all steps of the analysis, including mathematical formulae. This description should cover, as relevant, data cleaning, data pre-processing, data adjustments and weighting of data sources, and mathematical or statistical model(s).
11	Describe how candidate models were evaluated and how the final model(s) were selected.
12	Provide the results of an evaluation of model performance, if done, as well as the results of any relevant sensitivity analysis.
13	Describe methods for calculating uncertainty of the estimates. State which sources of uncertainty were, and were not, accounted for in the uncertainty analysis.
14	State how analytic or statistical source code used to generate estimates can be accessed.
Results and discussion	
15	Provide published estimates in a file format from which data can be efficiently extracted.
16	Report a quantitative measure of the uncertainty of the estimates (e.g. uncertainty intervals).
17	Interpret results in light of existing evidence. If updating a previous set of estimates, describe the reasons for changes in estimates.
18	Discuss limitations of the estimates. Include a discussion of any modelling assumptions or data limitations that affect interpretation of the estimates.

Application of the GATHER principles is likely to foster increased understanding of the technical underpinnings of global health estimates and contribute to more balanced dialogue between data producers and users and between international agencies and country decision makers, as called for in the paper by Pisani and Kok in this series [49]. While this may result in greater acceptance of global health estimates, the fact remains that they may give a misleading impression of certainty about country health trends. In the final analysis, global health estimates cannot replace the generation at subnational and national levels of high-quality data and statistics.

The international development community has called for investments in strengthening country health information and statistical systems [50–53]. Despite good intentions, initiatives have tended to be short-lived and sustained support to countries has remained limited. Modest investments in data collection have rarely been matched by resource allocation to country institutional capacity development for data quality assurance, data adjustment, and reconciliation techniques, and locally relevant analytical skills that are the bread and butter of high-quality national health statistics.

There are some grounds for optimism, however, with the launch in 2015 of the Data for Health Initiative [54], and the establishment of the Health Data Collaborative (HDC) [55]. The HDC is a joint commitment by multiple global health partners to align their financial and technical resources around a common agenda for measurement and accountability. Actions include strengthening governance of health and statistics systems; innovations in birth and death registration; routine use of verbal autopsy to ascertain causes of death in out-of-hospital settings; methods for data quality assessment; and support to building country capacities for review, analysis, interpretation and communication of data. With stronger information and statistical systems countries will be better placed to understand, critique and make appropriate use of global health estimates.

**Box 1.** Rationale for the production of global health estimates.

Completeness
To produce statistics for all countries for the same year using standardized methods.
To fill gaps, missing values in available data: information is generally available only for some countries and/or dates.
To generate comprehensive assessments of the burden of disease (GBD) to highlight priority health challenges.

Comparability
To deal with biases in the data; biases differ from place to place and may change over time within a country.
To ensure temporal and international comparability using similar methodology and assumptions across countries.
To reconcile differences between data sources and/or estimation method(s) for a specific data item and within sources over time.
Currency
To produce data of immediate or current relevance.
To respond quickly to demands for key indicators to meet demands for accountability and performance-based funding.
Cost
To generate the needed estimates in an inexpensive and rapid way that is not dependent on long-term capacity development efforts.
Objectivity
To ensure that country statistics are generated independently of political pressures.
To underpin accountability for results.

**Box 2.** International uses of global health estimates.

Tracking progress towards agreed goals and targets in countries and internationally.
Benchmarking progress against performance of socioeconomic or regional ‘peers’.
Informing results-based resource allocation.
Reporting programme performance to international agencies, donors, funds and foundations.
Identifying emerging international health priorities.
Generating interest in and advocating for programmes.
Providing comprehensive, comparable, internally consistent estimates of the burden of disease (and of risk factors).

**Box 3.** Country uses of global health estimates.

Identifying emerging health trends to be considered in national policy development.
Benchmarking country data and comparing progress with peers.
Monitoring trends over time and across subnational administrative areas matter more than cross-country comparisons.
Drawing attention to data deficiencies.
Highlighting and advocating for a health issue, e.g. NCDs, especially when country data are unavailable.
**But** only rarely for national planning and monitoring.

## Acronyms

BODBurden of disease

CHERGChild Health Epidemiology Reference Group

DHSDemographic and Health Surveys

GBDGlobal Burden of Disease

HDCThe Health Data Collaborative

IHMEInstitute for Health Metrics and Evaluation

MDGsMillennium Development Goals

MICSMultiple Indicator Cluster Surveys

SDGsSustainable Development Goals

UHCUniversal Health Coverage

UNFPAUnited Nations Population Fund

UNICEFUnited Nations Children’s Fund

UNPDUnited Nations Population Division

UNSDUnited Nations Statistics Division

USAIDUnited States Agency for International Development

WHOWorld Health Organization

## Paper context

Tracking progress towards development goals such as the MDGs and SDGs is heavily reliant on global health estimates developed by UN agencies and academics. While of value for development agencies on the grounds of comparability across countries and over time, they are of limited interest in countries, because they often conflict with locally generated data and are hard to explain to policy makers. This paper presents the differing perspectives of external producers of estimates and country users. It calls for greater transparency in estimation methods, increased involvement of countries in estimate development and meaningful and long term investments in building country capacities for data production and use.

## References

[1] Booth H, Gerland P. Demographic techniques: data adjustment and correction In: Wright JD, editor-in-chief. International Encyclopedia of the social & behavioral sciences. 2nd ed. Vol. 6 Oxford: Elsevier; 2015 p. 126–17.

[2] Declaration of Alma-Ata International conference on primary health care, Alma-Ata, USSR. Geneva: World Health Organization; 1978 9 6-12.

[3] World Health Organization Development of Indicators for Monitoring Progress towards Health for all by the year 2000. WHO 1981 9241800046. 1981 Available from: http://www.who.int/iris/handle/10665/40672

[4] World Health Organization World Health Statistics 2016: Monitoring Health for the SDGs, Sustainable Development Goals. 2016 ISBN 978 92 4 156526 4 (NLM classification: WA 900.1) E-ISBN 978 92 4 069569 6 (PDF) Available from: http://www.who.int/gho/publications/world_health_statistics/2016/EN_WHS2016_TOC.pdf?ua=1

[5] GBD 2015 SDG Collaborators Measuring the health-related sustainable development goals in 188 countries: a baseline analysis from the Global Burden of Disease Study 2015. Lancet. 2016;388:1813–1850. DOI:10.1016/S0140-6736(16)31467-2 27665228 PMC5055583

[6] Constitution of the World Health Organization. Official records of the World Health Organization No. 2 summary reports on proceedings, minutes and final acts of the international health conference; 1946 June 19–July 22. New York: United Nations.

[7] United Nations Demographic Yearbook 2014. New York (NY): Department of Economic and Social Affairs; 2015 ST/ESA/STAT/SER.R/44 Available from: http://unstats.un.org/unsd/demographic/products/dyb/dyb2.htm

[8] World Health Organization. Global Health Observatory. 2017. Available from: http://www.who.int/gho/en/

[9] World Health Organization World Health Statistics 2016: Monitoring Health for the SDGs, Sustainable Development Goals. 2016 Available from: http://www.who.int/gho/publications/world_health_statistics/2016/en/

[10] Rudan I, Campbell H, Marušić A, et al Assembling GHERG: could “academic crowd–sourcing” address gaps in global health estimates? J Glob Health. Available from: http://doi.org/10.7189/jogh.05.010101 10.7189/jogh.05.010101 PMC4593291 26445671

[11] Child Mortality Estimates [cited Jun 2015]. Available from: http://www.childmortality.org/index.php?r=site/graph

[12] World Health Organization World Health Statistics 2013. 2013 p. 5. Available from: http://www.who.int/gho/publications/world_health_statistics/2013/en/

[13] World Health Statistics Geneva: World Health Organization; 2016. p. 5.

[14] Vandermortele V. The MDG story: intention denied. In Development and Change. 2011;42:1–21. International Institute of Social Studies Blackwell Publishing, Malden, UK. Available from: http://onlinelibrary.wiley.com/doi/10.1111/dech.2011.42.issue-1/issuetoc

[15] Shiffman J Has donor prioritization of HIV/AIDS displaced aid for other health issues? Health Policy Plan. 2008;23:95–100. DOI:10.1093/heapol/czm045 18156161

[16] World Health Organization The World Health Report 2000: health systems: improving performance. Geneva: WHO; 2000 Available from: http://www.who.int/whr/2000/en/whr00_en.pdf

[17] McKee M The World Health Report 2000: 10 years on. Health Policy Plan. 2010;25:346–348. 20798126 10.1093/heapol/czq032

[18] World Health Organization The utility of estimates for health monitoring and decision-making: global, regional and country perspectives. Report of a technical meeting WHO. Glion sur Montreux (Switzerland): 2015 6 24–25 WHO/HIS/HSI/2015.7 Available from: https://extranet.who.int/iris/restricted/handle/10665/182163?locale=fr&null

[19] Alkema L, You D Child mortality estimation: a comparison of UN IGME and IHME estimates of levels and trends in under-five mortality rates and deaths. Plos Med. 2012;9:e1001288 DOI:10.1371/journal.pmed.1001288 22952434 PMC3429386

[20] Boerma T, Mathers C The World Health Organization and global health estimates: improving collaboration and capacity. BMC Med. 2015;13 DOI:10.1186/s12916-015-0286-7. PMC4355571 25858025

[21] Murray CLJ Towards good practice for health statistics: lessons from the Millennium development goal health indicators. Lancet. 2007;369:862–873. Available from: http://www.thelancet.com/journals/lancet/article/PIIS0140-6736(07)60415-2/abstract 17350457 10.1016/S0140-6736(07)60415-2 PMC7137868

[22] Bundhamcharoen K, Limwattananon S, Kusreesakul K, et al Contributions of national and global health estimates to monitoring health-related SDGs. 2016. 10.3402/gha.v9.32443 PMC5124116 28532308

[23] Sankoh O Global health estimates: stronger collaboration needed with low- and middle- income countries. Plos Med. 2010;7:e1001005 DOI:10.1371/journal.pmed.1001005 21151349 PMC2994665

[24] The PLoS Medicine Editors Can we count on global health estimates? Plos Med. 2010;7:e1001002 DOI:10.1371/journal.pmed.1001002 21151345 PMC2994661

[25] Walker N, Bryce J, Black RE Interpreting health statistics for policymaking: the story behind the headlines. Lancet. 2007 3 17;369:956–963. Review. PMID: 17368157 Available from: http://www.thelancet.com/journals/lancet/article/PIIS0140-6736(07)60454-1/abstract 17368157 10.1016/S0140-6736(07)60454-1

[26] Aguilera XP, Espinosa-Marty C, Castillo-Laborde C, et al From instinct to evidence: the role of data in country decision-making in Chile. 2016. 10.3402/gha.v9.32611 PMC5124120 28532306

[27] Mahy M, Brown T, Stover J, et al 2016 Producing HIV estimates: From global advocacy to country planning and impact measurement.

[28] http://cherg.org/about/partners.html.

[29] Murray CJL, Lopez AD, Wibulpolprasert S Monitoring global health: time for new solutions. BMJ. 2004;329:1096–1100. DOI:10.1136/bmj.329.7474.1096 15528624 PMC526127

[30] Institute for Health Metrics and Evaluation The global burden of disease: generating evidence, guiding policy. Seattle (DC): Institute for Health Metrics and Evaluation; 2013 Available from: http://www.healthdata.org/policy-report/global-burden-disease-generating-evidence-guiding-policy

[31] GBD 2015 Mortality and Causes of Death Collaborators. Global, regional, and national life expectancy, all-cause mortality, and cause-specific mortality for 249 causes of death, 1980–2015: a systematic analysis for the Global Burden of Disease Study 2015. Lancet. 2016;388:1459–1544. 10.1016/S0140-6736(16)31012-1 PMC5388903 27733281

[32] World Bank. World development report 1993: investing in health. New York: Oxford University Press; 1993. Available from: https://openknowledge.worldbank.org/handle/10986/5976 License: CC BY 3.0 IGO..

[33] Institute for Health Metrics and Evaluation The Global Burden of Disease: a Critical Resource for Informed Policymaking. 2016 Available from: http://www.healthdata.org/gbd

[34] Anand S, Hanson K Disability adjusted life years: a critical perspective. J Health Econ. 1997;16:685–702. Available from: http://www.sciencedirect.com/science/article/pii/S0167629697000052 10176779 10.1016/s0167-6296(97)00005-2

[35] Byass P, De Courten M, Graham WJ, et al Reflections on the global burden of disease 2010 estimates. Plos Med. 2013;10:e1001477 DOI:10.1371/journal.pmed.1001477 23843748 PMC3699446

[36] Parks R The rise, critique and persistence of the DALY in global health. J Glob Health. 2014 Available from: http: //www.ghjournal.org/the-rise-critique-and-persistence-of-the-daly-in-global-health/

[37] Lozano R, Naghavi M, Foreman K, et al Global and regional mortality from 235 causes of death for 20 age groups in 1990 and 2010: A systematic analysis for the Global Burden of Disease Study 2010. Lancet. 2012;380:2095–2128. http://www.thelancet.com/journals/lancet/article/PIIS0140-6736(12)61728-0/abstract 23245604 10.1016/S0140-6736(12)61728-0 PMC10790329

[38] Ruano AL, Friedman EA, Hill PS Health, equity and the post-2015 agenda: raising the voices of marginalized communities. Int J Equity Health. 2014;13:82 Available from: http://www.equityhealthj.com/content/13/1/82 25300905 10.1186/s12939-014-0082-6 PMC4201725

[39] Hoffman S Citizen science: the law and ethics of public access to medical big data. Berkeley Technol Law J. Forthcoming 2014 [cited 2015 9 9]. Available from: http://papers.ssrn.com/sol3/papers.cfm?abstract_id=2491054

[40] United Nations Department of Economic and Social Affairs Statistics Division Fundamental Principles of Official Statistics. 2006 Available from: http://unstats.un.org/unsd/dnss/gp/fundprinciples.aspx

[41] Gliklich RE, Dreyer NA, Leavy MB, editors. Registries for evaluating patient outcomes: a user's guide [Internet]. 3rd ed. Rockville (MD): Agency for Healthcare Research and Quality (US); 2014 Apr 7. Principles of Registry Ethics, Data Ownership, and Privacy. Available from: https://www.ncbi.nlm.nih.gov/books/NBK208620/ 24945055

[42] Willyard C Electronic records pose dilemma in developing countries. Nat Med. [Internet] 2010; 16: 249 [cited 2015 9 9] Available from: http://www.nature.com/nm/journal/v16/n3/full/nm0310-249a.html 10.1038/nm0310-249a 20208497

[43] Sixty-sixth world health assembly WHA66.24 Agenda item 17.5 27 May 2013 eHealth standardization and interoperability. Geneva: World Health Organization; 2013. Available from: http://www.who.int/topics/ehealth/en/

[44] World Health Organization mHealth: new horizons for health through mobile technologies: second global survey on eHealth. Global Observatory for Ehealth. 2011 Available from: http://www.who.int/goe/publications/goe_mhealth_web.pdf?ua=1

[45] World Health Organization Global health estimates: proposals on the way forward. Summary of a Technical Meeting WHO; 2013 2 13-14; Geneva Available from: http://www.who.int/healthinfo/GHE_MeetingSummary_Feb2013.pdf?ua=1

[46] The utility of estimates for health monitoring and decision-making: global, regional and country perspectives. Report of a technical meeting. Glion sur Montreux, Switzerland Available from: http://apps.who.int/iris/bitstream/10665/182163/1/WHO_HIS_HSI_2015.7_eng.pdf?ua=1&ua=1

[47] Stevens GA, Alkema L, Black RE, et al. Correction: guidelines for accurate and transparent health estimates reporting: the GATHER statement. PLoS Med 13(8):e1002116. doi:10.1371/journal.pmed.1002116 PMC4978408 27504831

[48] Cokljat M, Henderson J, Paterson A, et al Reporting of health estimates prior to GATHER: a scoping review. 2016. 10.1080/16549716.2017.1267958 PMC5645696 28532309

[49] Pisani E, Kok M In the eye of the beholder: to make global health estimates useful, make them socially robust. 2016. 10.3402/gha.v9.32298 PMC5124117 28532303

[50] Laliberté L Statistical Capacity Building Indicators Final Report. PARIS21 Task Team on Statistical Capacity Building Indicators. 2002 Available from: http://www.paris21.org/sites/default/files/scbi-final-en.pdf

[51] Paris 21 A Guide to Designing a National Strategy for the Development of Statistics (NSDS). Paris 2004. 2004 Available from: http://siteresources.worldbank.org/SCBINTRANET/Resources/NSD_Guide-Nov04.pdf

[52] Health Metrics Network & WHO Framework and standards for country health information systems. 2nd ed. Geneva: World Health Organization; 2008 Available from: http://apps.who.int/iris/bitstream/10665/43872/1/9789241595940_eng.pdf

[53] Strengthening health information systems in the Asia-Pacific region aims, objectives and contributions of the Health information systems knowledge hub at the University of Queensland, 2008-2013, Brisbane, Australia. Brisbane, Australia: University of Queensland; 2013 Available from: http://www.uq.edu.au/hishub/

[54] Bloomberg Philanthropies. Data for Health Initiative. 2015. Available from: https://www.bloomberg.org/program/public-health/data-health/

[55] The Health Data Collaborative. 2015. Available from: http://www.healthdatacollaborative.org/what-we-do/

